# Evidence of inhibin/activin subunit betaC and betaE synthesis in normal human endometrial tissue

**DOI:** 10.1186/1477-7827-8-143

**Published:** 2010-11-19

**Authors:** Ioannis Mylonas, Ansgar Brüning, Naim Shabani, Susanne Kunze, Markus S Kupka

**Affiliations:** 1 Ludwig-Maximilians-University Munich, 1st Department of Obstetrics and Gynecology, Maistrasse 11, 80337 Munich, Germany; 2 Department of Obstetrics and Gynecology, Klinikum Neuperlach, Munich, Germany

## Abstract

**Background:**

Inhibins are important regulators of the female reproductive system. Recently, two new inhibin subunits betaC and betaE have been described, although it is unclear if they are synthesized in normal human endometrium.

**Methods:**

Samples of human endometrium were obtained from 82 premenopausal, non-pregnant patients undergoing gynecological surgery for benign diseases. Endometrium samples were classified according to anamnestic and histological dating into proliferative (day 1-14, n = 46), early secretory (day 15-22, n = 18) and late secretory phase (day 23-28, n = 18). Immunohistochemical analyses were performed with specific antibodies against inhibin alpha (n = 81) as well as inhibin betaA (n = 82), betaB (n = 82), betaC (n = 74) and betaE (n = 76) subunits. RT-PCR was performed for all inhibin subunits. Correlation was assessed with the Spearman factor to assess the relationship of inhibin-subunits expression within the different endometrial samples.

**Results:**

The novel inhibin betaC and betaE subunits were found in normal human endometrium by immunohistochemical and molecular techniques. Inhibin alpha, betaA, betaB and betaE subunits showed a circadian expression pattern, being more abundant during the late secretory phase than during the proliferative phase. Additionally, a significant correlation between inhibin alpha and all inhibin beta subunits was observed.

**Conclusions:**

The differential expression pattern of the betaC- and betaE-subunits in normal human endometrial tissue suggests that they function in endometrial maturation and blastocyst implantation. However, the precise role of these novel inhibin/activin subunits in human endometrium is unclear and warrants further investigation.

## Background

Together with bone morphogenetic proteins (BMPs), growth and differentiation factors (GDFs), myostatin, Muellerian inhibiting substance (MIS) and other proteins [1-3], inhibin and activin proteins belong to the transforming growth factor-beta (TGF-β) family of growth and differentiation factors. Within this inhibin subgroup, one α-subunit and four β-subunit isoforms (βA, βB, βC and βE) have been isolated in mammals and humans [2-6]. The β-subunits can form activins by dimerization with a second β-subunit, or alternatively, they can form inhibins by dimerizing with an α-subunit. Thus, depending on the subunit combination, there are two isoforms of inhibin (inhibin A (α-βA) and inhibin B (α-βB)) and three isoforms of activin (activin A (βA-βA), activin B (βB-βB) and activin AB (βA-βB)) [2,3]. Recently, two additional β-subunits were identified in humans, βC [4] and βE [6]. These two novel subunits share 82% and 61% amino acid sequence similarity with the corresponding mature proteins from rat and mouse, respectively [7,8].

Inhibins and activins were initially isolated from the gonads and have been demonstrated to be disulfide-linked dimers [1-3]. Meanwhile, the well-studied inhibin α-, βA-, and βB-subunits have been detected in normal and abnormal endometrial tissue [9-15], and are implicated as important paracrine modulators of reproductive function [16,17] and malignant transformation [12,13,18]. Moreover, inhibins and activins might play an important role in endometrial cell function by regulating endometrial maturation, decidualization, and human implantation processes [19-26].

However, only limited data on histological expression of the inhibin/activin βC and βE subunits in normal human endometrium are available. The inhibin βC protein was primarily expressed in human liver and prostate [27], while inhibin/activin βE mRNA was predominantly synthesized in human liver with low levels found in heart, testis, leukocytes, and skeletal muscle [5]. Inhibin βC was previously detected by immunohistochemical methods in normal and abnormal placenta [28,29], endometrial cell lines [30,31], endometrial cancer [32], and cervical tissue [33]. Additionally, the inhibin βE is synthesized in normal and abnormal placenta [5,34,35] as well as human cervical tissue [36] and the endometrial cancer cell line Ishikawa [31].

Since specific monoclonal antibodies against inhibin subunits are only recently available, systematic investigations on the combined expression of inhibin/activin subunits have not been performed. Uncovering the differential expression patterns of the five inhibin/activin subunits and their correlations in human endometrium will further understanding of human reproduction. Additionally, knowledge of the expression patterns of the inhibin β-subunits is important, since activin signaling might be a promising target for therapeutic interventions [37].

## Methods

### Tissue samples

Immunohistochemical analysis of inhibin-subunits was performed on a well-characterized patient group [11,38]. Samples of human endometrium were obtained from 82 premenopausal, non-pregnant patients undergoing gynecological surgery for benign diseases (mainly uterine leiomyoma) either by D&C (dilatation and curettage) or hysterectomy. We had recently analyzed 54 endometrial samples for the expression of inhibin-α, -βA and -βB [11], that have been included in this study. All women had a normal and regular menstrual cycle with no hormonal treatment for 3 months prior to surgery. All pathological and hyperplastic endometrial samples were excluded from this study. Endometrium samples were classified according to anamnestic and histological dating into proliferative (day 1-14, n = 46), early secretory (day 15-22, n = 18) and late secretory phase (day 23-28, n = 18) as previously described [11,38-40].

### Generation of a polyclonal inhibin-βE peptide antibody

Anti-inhibin βE polyclonal antibodies were generated as custom-made antibodies in rabbits against a polypeptide of 16 amino acids of inhibin βE (polypeptide-sequence: NH2-CRWGPRRRRQGSRTLL-COOH; amino acid position 144 to 158; accession number: AAH05161) as previously described by BioGenes (Berlin, Germany) [41]. This amino acid sequence is unique to the inhibin βE subunit and is absent from other inhibin subunits. This, as well as optimal immunogenic properties, was checked prior to immunization by bioinformatic programs (Antheprot; protein sequence alignments) [34].

A primary dose of 200 μg activin beta E polypeptide was solubilized in Freund's complete adjuvant (Sigma, Aldrich, Germany) and injected subcutaneously into rabbits. Three doses of the peptide solubilized in Freund's incomplete adjuvant were administrated at 6 week intervals. After the third booster injection (14 days), blood was collected from the rabbit, and the serum was separated. Antibodies were isolated by column chromatography with a protein A column (Amersham Pharmacia Biotech, Freiburg, Germany).

### Immunohistochemistry

Immunohistochemical analyses were performed using a combination of heat induced antigen retrieval and the standard streptavidin-biotin-peroxidase complex using the mouse (for the inhibin α, βA and βB antibodies), goat (for inhibin βC antibody) or rabbit (for inhibin βE antibody) IgG-Vectastain Elite ABC kit (Vector Laboratories, Burlingame, California, USA) as previously described [11,31,34,36,41].

Antibodies used for these experiments are listed in Table 1. The immunohistochemical procedures for inhibin α-, βA- and βB-subunits were evaluated in normal and malignant endometrial tissue [11-13,42,43], breast cancer tissue [44], and uterine cervical cancer [45] as well as normal and abnormal placenta [46,47]. The inhibin βC antibody was previously evaluated in normal and pathological placenta tissue [28,29], endometrial cell lines [30,31], endometrial cancer [32] and cervical tissue [33]. The immunohistochemical procedure for inhibin βE was performed in ovarian tissue [41], endometrial cancer cell lines [31], normal and abnormal placenta [34], and uterine cervical tissue [36].

**Table 1:** Antibodies used for immunohistochemical characterization of human endometrium

Antibody	Clone	Isotype	Dilution	Dilution medium	Source
**Inhibin-α**	R1	Mouse IgG2a	1:50	PBS	Serotec, Oxford, UK
**Inhibin-βA**	E4	Mouse IgG2b	1:50	PBS	Serotec, Oxford, UK
**Inhibin-βB**	C5	Mouse IgG2a	1:10	PBS	Serotec, Oxford, UK
**Inhibin-βC**	Polyclonal	Goat IgG	1:50	Ultra-V-Block	R&D Systems, Wiesbaden, Germany
**Inhibin-βE**	Polyclonal	Rabbit IgG	1:4000	Ultra-V-Block	BioGenes, Berlin, Germany

Briefly, paraffin-fixed tissue sections were dewaxed using xylol for 15 min, rehydrated in descending series of alcohol (100%, 96% and 70%), and subjected to antigen retrieval for 10 min in a pressure cooker using sodium citrate buffer (pH 6.0), containing 0.1 M citric acid and 0.1 M sodium citrate in distillated water. After reaching room temperature, sections were washed twice in phosphate-buffered saline (PBS). Endogenous peroxidase activity was quenched by immersion in 3% hydrogen peroxide (Merck, Darmstadt, Germany) in methanol for 20 min. Non-specific binding of the primary antibodies was blocked by incubating the sections with diluted normal serum (10 ml PBS containing 150 μl horse serum; provided by Vector Laboratories) for 20 min at room temp. Sections were then incubated at room temperature for 60 min with the primary antibodies for inhibin-α, -βA and -βB. Inhibin-α, -βA and -βB were diluted in PBS. For inhibin-βC and -βE antibodies, sections were incubated at 4C over night with the inhibin-βC polyclonal goat antibody at a dilution of 1:50 in Ultra-V-Block (Lab Vision, Fremont, U.S.A.) or the inhibin-βE polyclonal rabbit antibody at a dilution of 1:4000 in Ultra-V-Block (Lab Vision, Fremont, U.S.A.). After washing with PBS, sections were incubated in diluted biotinylated serum (10 ml PBS containing 50 μl horse serum; provided by Vector Laboratories) for 30 min at room temperature. After incubation with the avidin-biotin peroxidase complex (diluted in 10 ml PBS; Vector Laboratories) for 30 min and repeated washing steps with PBS, visualization was performed with substrate and chromogen 3,3'-diaminobenzidine (DAB; Dako, Glostrup, Denmark) for 8-10 min. Sections were counterstained with Mayer's acidic hematoxylin and dehydrated in an ascending series of alcohol (50-98%). After xylol treatment, sections were covered. Negative controls were performed by replacing the primary antibody with normal mouse, rabbit or goat IgG as isotype control in the same dilution compared to the primary antibody, respectively. Immunohistochemical staining was performed using an appropriate positive control comprising ovaries containing follicular cysts [11,31,41]. Positive cells showed a brownish color and negative controls as well as unstained cells were blue. Sections were examined using a Leitz (Wetzlar, Germany) photomicroscope. Digital images were obtained with a digital camera system (JVC, Japan) and were saved on computer (Diskus software, Hilgers, Königswinter, Germany).

### RT-PCR analysis

To analyze inhibin subunit expression at the transcriptional level, RNA was extracted from samples of human endometrium tissue of proliferative (n = 3) and secretory phase (n = 3) cells, transcribed into cDNA, and analyzed by RT-PCR analysis for the expression of inhibin subunits α, βA, βB, βC and βE using specific primers (Table 2).

**Table 2:** Primer sequences and length of amplification products

	Forward primer (5' - 3')	Backward primer (3' - 5')	Length
**Inhibin-α**	CCGGCCATCCCAGCATACACGC	GAGTTGAGCGTCGGGCTCTC	359 bp
**Inhibin-βA**	TGCCCTTGCTTTGGCTGAGA	ACTTTGCCCACATGAAGCTTT	282 bp
**Inhibin-βB**	GGCGAGCGGCGACTCAACCTAGA	CGTGTGGAAGGAGGAGGCAGAGC	333 bp
**Inhibin-βC**	GCAGCCCGGGTGAGAGTTGG	ACTGCACCCACAGGCCTC	393 bp
**Inhibin-βE**	AGCCCTTCCTAGAGCTTAAG	GCTGCAGCCACAGGCC	404 bp

bp = base pairs

RNA was extracted from cells using the Nucleospin RNA II kit (Macherey-Nagel, Düren, Germany) as previously described [29,31,34,36]. Reverse transcription was performed with M-MLV reverse transcriptase and oligo-dT (Promega, Mannheim, Germany) as recommended by the supplier. PCR was performed in an Eppendorf Mastercycler with GoTaq (Promega, Mannheim, Germany). Primer sequences are listed in Table 2. PCR cycling was performed after a 5 min initiation at 94°C with 36 cycles of 1 min at 94°C, 1 min at 57°C, 2 min at 72°C, followed by a 5 min extension at 72°C. For a cDNA quality control, actin primers (Stratagene, The Netherlands), amplifying a 661 bp product, were used. As a further control, cDNA was omitted (water control) to present any PCR contaminations [29]. PCR products were separated on a 1.5% agarose gel, including a pBR328 marker (Roth, Karlsruhe, Germany). Gels were stained with SYBR Safe (1:10,000 dilution; Invitrogen, Karlsruhe, Germany) prior to gel electrophoresis and after a completed run transferred on a UV-permeable tray to a BioRad Image Analyser (BioRad, Munich, Germany) [29]. The generated electronic picture file was exported as a TIFF file and imported in a Power-Point presentation file in order to crop and label the figure.

### Evaluation and statistical analysis

The intensity and distribution patterns of specific inhibin-α, -βA,- βB, -βC and -βE subunit immunohistochemical staining was evaluated using the semi-quantitative score (IRS) as previously described and used to asses inhibin/activin subunits [11,13]. Briefly, the IRS score was calculated by multiplication of optical staining intensity (graded as 0 = no, 1 = weak, 2 = moderate and 3 = strong staining) by the percentage of positive stained cells (0 = no staining, 1 = <10% of the cells, 2 = 11-50% of the cells, 3 = 51-80% of the cells and 4 = >81% of the cells). The slides were examined by two independent observers, including a gynecological pathologist (N.S.). Sections were examined using a Leitz (Wetzlar, Germany) photomicroscope. Digital images were obtained with a digital camera system (JVC, Yokohama, Japan) and were saved on computer (Diskus software, Hilgers, Königswinter, Germany). The results were evaluated using the non-parametric Mann-Whitney U rank-sum test (SPSS, Version 17.0, Chigaco, IL, USA). Correlation was assessed with the Spearman factor to assess the relationship of inhibin-subunits expression within the different endometrial samples. Significance was assumed at p < 0.05 by using the two-tailed test.

## Results

### Immunohistochemical expression of inhibin-α, -βA, -βB, -βC and -βE

**Figure 1:**
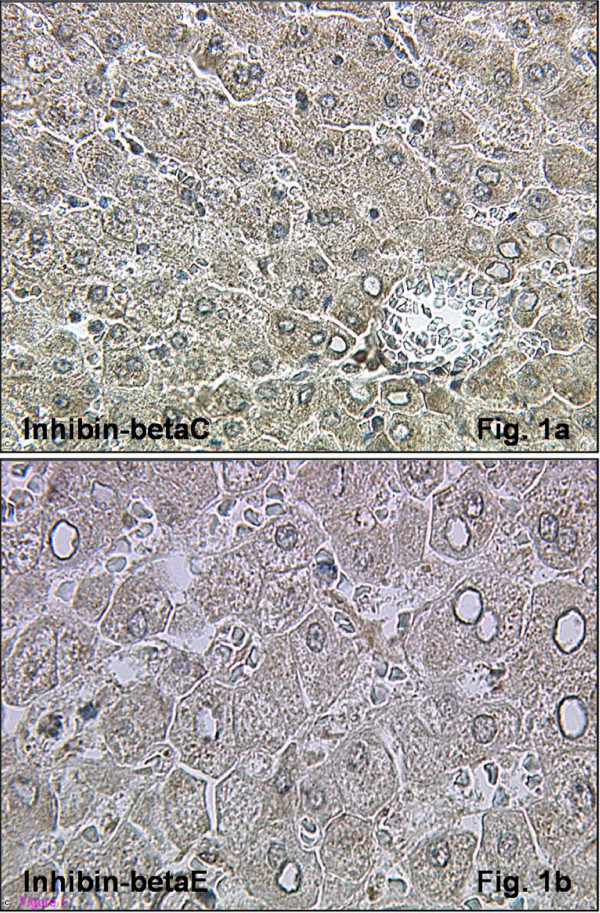
**Immunohistochemical expression of inhibin-βC and -βE in normal liver tissue**. Inhibin-βC (Figure 1a; magnification ×400) and βE (Figure 1b; magnification ×400) subunit demonstrated a positive staining reaction in normal human liver tissue.

To test the antibodies against the βC- and βE-subunits, evaluation of the immunohistochemical staining reaction was performed using appropriate positive controls, including normal liver specimens (Figure 1a-b) and previous results were confirmed [5,29,31,34,36,41,48]. The inhibin/activin α-, βA-, and βB-subunits were detected in normal human endometrial tissue throughout the menstrual cycle in accord with previous results (data not shown). Inhibin α, βA, and βB were primarily observed in glandular and luminal epithelial cells, with a different staining intensity in stromal cells, especially during secretory phase. Proliferative and secretory endometria expressed the βA- and βB-subunits with a similar pattern with the maximal immunostaining intensity observed in secretory phase tissues (data not shown) (Table 3) [11,15].

**Table 3:** Immunohistochemical findings determining localization and intensity of immunostaining intensity for inhibin-α, -βA,- βB, -βC and -βE subunits in human endometrium across the normal menstrual cycle

	Inhibin subunits	Proliferative phase	Early secretory phase	Late secretory phase
**Glandular epithelium**	α	±	+	++
	βA	+	+	++
	βB	+	+	++
	βC	+	+	+
	βE	+	+	++
**Stromal cells**	α	-	+	++
	βA	-	+	++
	βB	-	+	++
	βC	+	+	+
	βE	-	+	++

- = no staining; +/- = minimal staining + = positive staining; ++ = intense staining.

**Figure 2:**
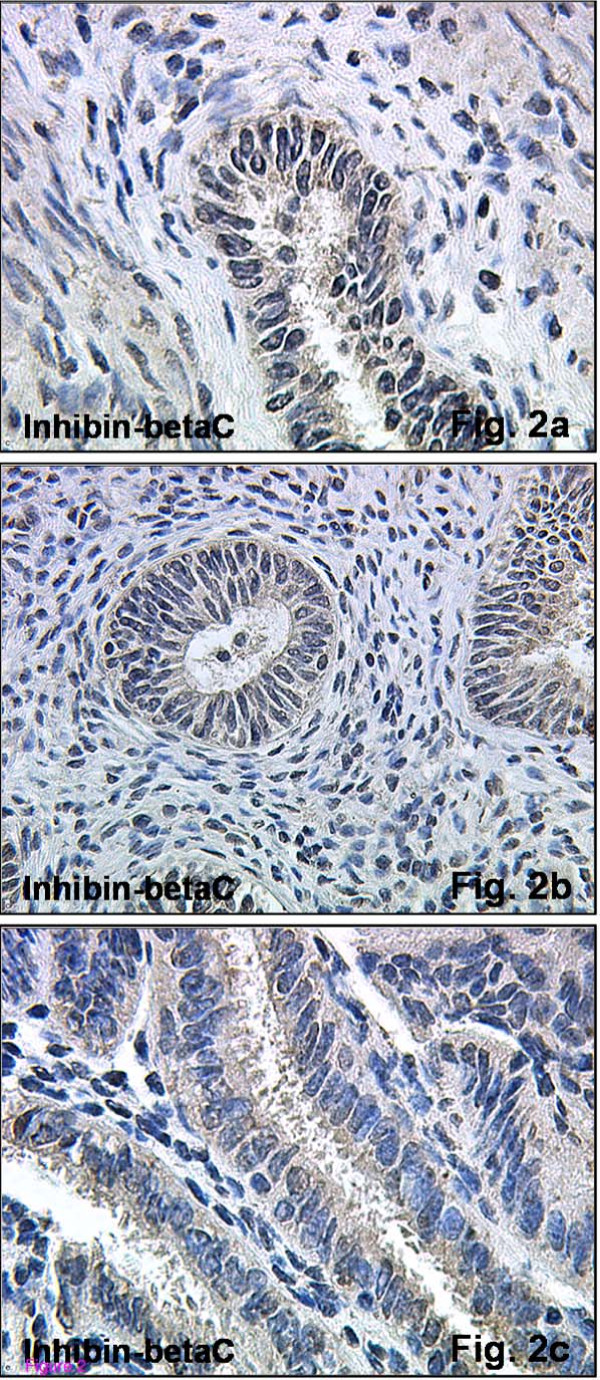
**Immunohistochemical expression of inhibin-βC in normal human endometrial tissue**. Immunohistochemical staining reaction for inhibin-βC could be observed across the menstrual cycle. Proliferative (Figure 2a, magnification ×250) and early secretory endometria (Figure 2b, magnification ×125) expressed this subunit, but with a weak intensity. The strongest staining intensity could be observed during tissue samples late secretory phase (Figure 2c, magnification ×400). Stromal cells also reacted positively throughout the menstrual cycle.

The βC-subunit was also detected in endometrial glandular epithelial cells. A diffuse immunohistochemical staining reaction was observed in cells representing all stages of the menstrual cycle. The staining intensity of cells was weaker during early secretory phase (Figure 2b) compared to the cells from endometrial samples from proliferative phase (Figure 2a), while the maximal immunostaining reaction was observed in cells isolated from late secretory phase tissues (Figure 2c). Interestingly, the βC-subunit immunoreactivity was detected in all stages of the menstrual cycle examined with little variation in intensity. (Figure 2c).

**Figure 3:**
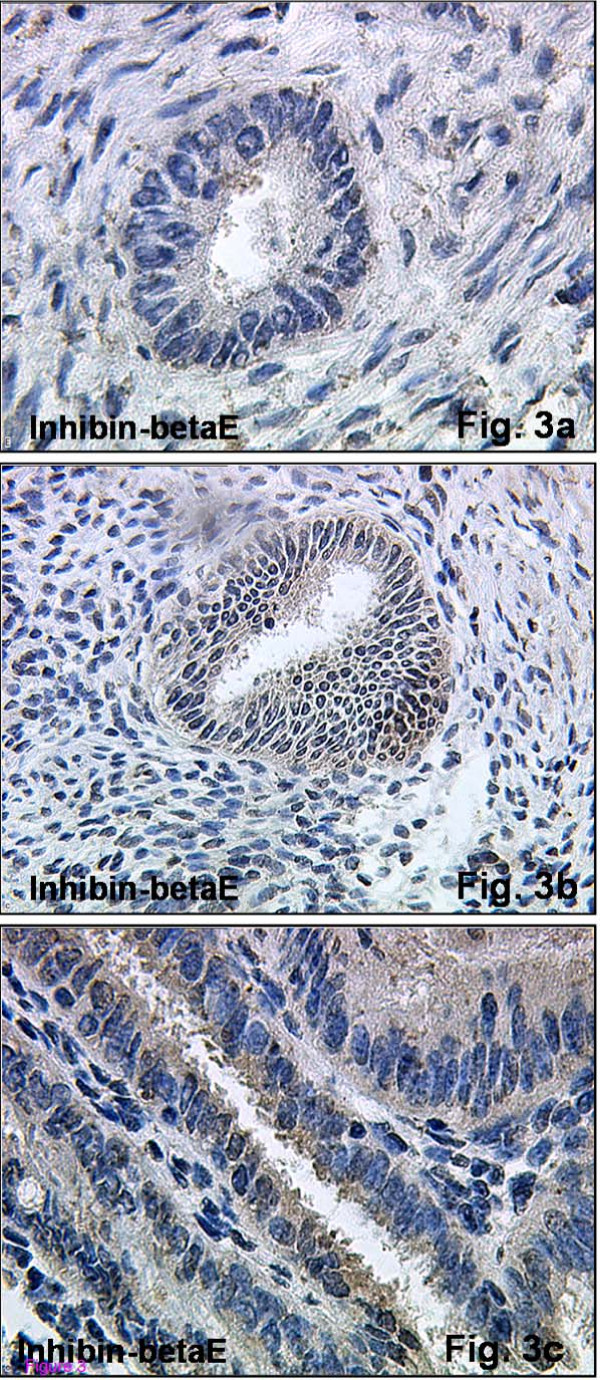
**Immunohistochemical expression of inhibin-βE in normal human endometrial tissue**. Immunohistochemical staining reaction for inhibin-βE could be observed across the menstrual cycle. Proliferative endometrial tissue (Figure 3a, magnification ×250) demonstrated a weak staining intensity, while this intensity increased during early (Figure 3b, magnification ×125) and late secretory endometria. The strongest staining intensity could be observed during tissue samples late secretory phase (Figure 3c, magnification ×400). Stromal cells also reacted positively with a minimal staining intensity in the proliferative phase, being moist prominent during late secretory phase.

The inhibin βE-subunit was also expressed in human endometrium primarily in the glandular and surface epithelium. During proliferative phase, minimal immunoreactivity was observed at the apical component of glandular cells (Figure 3a). The staining intensity increased in tissues from the early secretory phase, and was localized to the basal and apical components of the cells (Figure 3b). The strongest immunoreactivity was observed during late secretory phase (Figure 3c). With the onset of early secretory phase, the βE-subunit was also detected in stromal cells (Table 3) (Figure 3c). However, no statistically significant differences in immunoreactivity found in stromal cells was observed for inhibin βC and βE at any stage of the menstrual cycle (data not shown).

### RT-PCR analysis

**Figure 4:**
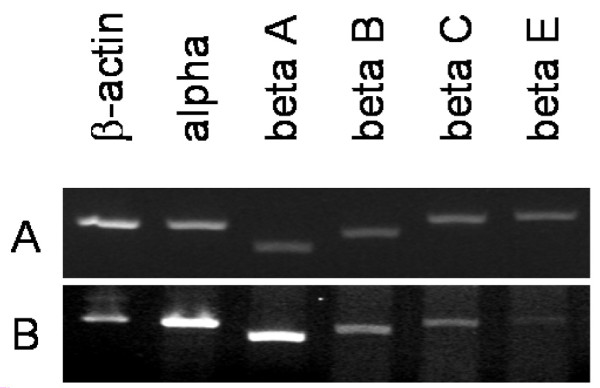
**Inhibin/activin-subunit mRNA detection in normal human endometrium tissue**. The mRNA of all inhibin/activin-subunits was detected in normal proliferative (Figure 4a) and secretory (Figure 4b) human endometrium using PCR.

Figure 4 demonstrates that the mRNA of all inhibin subunits is expressed in normal human endometrium tissue of the secretory phase (Figure 4a) as well as of the proliferative phase (Figure 4b). Overall, the expression of inhibin subunit mRNAs appeared to be higher in the secretory phase than in the proliferative phase, although the small sample number and activities of potential regulatory mechanisms at the post-transcriptional level did not allow further conclusions to be drawn.

### Statistical analysis

A statistically significant correlation between the immunoreactive score (IRS) of the inhibin α and all inhibin β subunits was observed by using the non-parametric Spearmen correlation factor (p < 0.05). However, no significant correlation between the amount of the βC-subunit and the amount of the other β-subunits was demonstrated. The IRS of the inhibin βE-subunit showed a significant association with the amount of the inhibin βB-subunit (p < 0.05) (Table 4).

**Table 4:** Correlation between all five inhibin-subunits

		Inhibin-α	inhibin-βA	inhibin-βB	Inhibin-βC	Inhibin-βE
**Inhibin-α**	Correlation Coefficient (r)		0,409	0,534	0,260	0,533
	*p*		*< 0,001*	*< 0,001*	*< 0,05*	*< 0,001*
	n		81	80	73	75
**inhibin-βA**	Correlation Coefficient (r)	0,409		0,539	0,097	0,209
	*p*	*< 0,001*		*< 0,001*	*N.S*.	*N.S*.
	n	81		81	74	76
**inhibin-βB**	Correlation Coefficient (r)	0,534	0,539		0,231	0,321
	*p*	*< 0,001*	*< 0,001*		*= 0,05*	*< 0,01*
	n	80	81		73	75
**Inhibin-βC**	Correlation Coefficient (r)	0,260	0,097	0,231		0,027
	*p*	*< 0,05*	*N.S*.	*= 0,05*		*N.S*.
	n	73	74	73	74	69
**Inhibin-βE**	Correlation Coefficient (r)	0,533	0,209	0,321	0,027	
	*p*	*< 0,001*	*< 0,05*	*< 0,01*	*N.S*.	
	n	75	76	75	69	

N.S. = not significant

**Figure 5:**
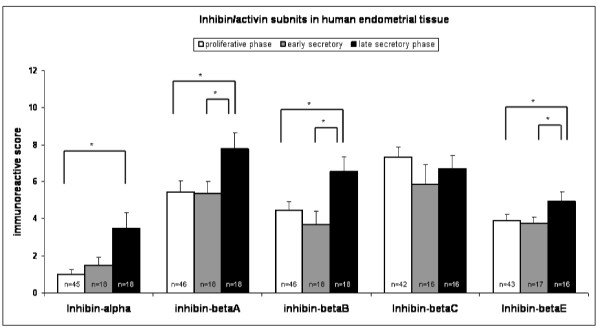
**Immunohistochemical score for the expression of all five analyzed inhibin/activin subunits**. The immunohistochemical staining reaction for inhibin-α was significantly lower during proliferative compared to late secretory phase. Moreover, the immunohistochemical expression of the inhibin-βA, -βB, and -βE were significantly higher during late secretory compared to proliferative and early secretory phase (*). Inhibin-βC, although being lower during early secretory phase, did not demonstrate any significant changes throughout the menstrual cycle. Values represent means ± SEM. Significance was assumed at p < 0.05 (asterisks).

The immunoreactive score for inhibin βA, βB and βE increased between proliferative and late secretory phase (p < 0.05) as well as between early and late secretory phase (p < 0.05) (Figure 5). Inhibin α expression was higher during late secretory phase compared to proliferative endometrial tissue (p < 0.05), while the immunoreactive score for inhibin βC did not demonstrate any changes during the menstrual cycle (Figure 5).

## Discussion

### Inhibin-subunits in human endometrium

Inhibins and activins were initially characterized as endocrine and paracrine hormonal regulators of the hypothalamic-pituitary-gonadal axis. Several autocrine and paracrine actions of inhibins/activins have been reported including modulation of ovarian and placental hormone secretion [17,21,49,50], decidualization [21-24,51], and trophoblast differentiation [20,52], as well as effects on immunomodulatory function [53], stem cell biology [54,55], and apoptosis [56]. However, these putative functions have been characterized for inhibin α, βA and βB isoforms, and it is not clear if the βC- and βE- isoforms have similar function.

Meanwhile, these proteins are expressed in a wide range of female reproductive tissues including normal and abnormal human endometrium [9,10,12,13,15,42,43]. In this study, a circadian expression of the well-known inhibin α-, βA-, and βB-subunits in normal human endometrial tissue was demonstrated, confirming previous results [10,11]. Moreover, expression of the novel inhibin/activin βC- and βE-subunits was also demonstrated in normal human endometrium by using RT-PCR and immunohistochemical detection methods. The βE-subunit demonstrated an increase in staining intensity between proliferative and late secretory phase similar to results with inhibin α, βA and βB immunolabeling. By contrast, the βC-subunit did not show any difference in immunohistochemical staining intensity between proliferative and secretory phase. A decrease of the inhibin βC-subunit staining intensity was demonstrated between proliferative and late secretory phase, but this was not statistically significant.

The inhibins α, βA and βB probably have important functions in blastocyst implantation and contribute to the paracrine signaling needed for adequate endometrial maturation [22]. The potential roles of the novel inhibin/activin subunits in human reproduction are intriguing. Results suggest that both βC- and βE-subunits also play an important role in the human menstrual cycle. Additionally, the distinct expression of inhibin-α in the apical part of the glandular epithelial cells shows the preferred synthesis of inhibins in the uterine lumen. Since expression is lower in the endometrial stroma, activins are probably produced and secreted into this compartment [11].

### Inhibin βC-subunit

Inhibin βC was primarily detected in hepatocytes [5,48] and is implicated in the regulation of liver cell growth as demonstrated by downregulation of inhibin βC mRNA after partial hepatectomy in rats [57,58]. The formation of homodimeric activin C (βC-βC), heterodimeric activins AC (βA-βC), BC (βB-βC), CE (βC-βE), and inhibin C (α-β) has been demonstrated by ectopic expression of the respective subunits in different cell models [27,59]. Although the precise role of the βC subunit has not been elucidated, several possible functions have been suggested. Interestingly, ectopic expression of inhibin/activin βC subunit induced apoptosis in human (HepG2, Hep3B) and rat (H4, EC3) hepatoma cells [60,61]. In an immortalized mouse hepatocyte cell line (AML12) and primary rat hepatocytes, activin βC increased the rate of DNA synthesis [62]. Moreover, the βC-subunit was identified as an autocrine growth modulator in liver regeneration, leading to mitosis in a subset of hepatocytes [63].

In human reproduction, this βC subunit is not believed to be a significant regulator of activin bioactivity, since no abnormalities or malformations have been observed in inhibin-βC knockout mice [48]. However, there might be functional redundancy with other TGF-β factors [64] and an antagonistic and regulatory role for activin A bioactivity has recently been proposed [27,64,65]. Interestingly, activin C (βC-βC) did not activate activin A (βA-βA) responsive promoters, and it was suggested that the βC-subunit regulates the levels of bioactive activin A (βA-βA) through the formation of signaling incompetent activin AC heterodimers [64-66].

A preliminary study using RT-PCR and immunohistochemical detection methods demonstrated expression of inhibin βC in normal human endometrium and the human endometrial carcinoma cell line Ishikawa [31]. The lower expression observed during early secretory phase compared to that found in the proliferative phase might suggest a mitotic function [63]. Thus, inhibin βC might participate in the proliferation of human endometrium during the first phase of the menstrual cycle and reduce the mitotic effect during blastocyst implantation. However, no significant differences in βC-subunit expression during the menstrual cycle were found. This might be attributed either to the antibody used or to the small number of samples analyzed. In addition, the expression pattern of this subunit is different from the other β-subunits, and this suggests that it may have a different function in human endometrium.

### Inhibin βE-subunit

Similar to the βC-subunit, the βE-subunit is predominantly expressed in hepatocytes, although it is also detected in human heart, testis, peripheral blood leucocytes, and skeletal muscle tissue [5,6,61,67]. Moreover, inhibin βE is synthesized in normal and abnormal placenta [5,34,35] and human cervix [36]. This novel subunit was also demonstrated in the Ishikawa endometrial carcinoma cell line, which suggests that these cells may be useful as an *in vitro *model for studying the βE-subunit [31].

Formation of homodimeric activin E as well as heterodimeric activins AE (βA-βE) and CE (βC-βE) has been demonstrated after ectopic co-expression of these respective subunits [5,8]. Interestingly, inhibin/activin βE mRNA expression was transiently upregulated after partial hepatectomy or portal vein branch ligation [48,68]. When ectopically expressed in HepG2 or Hep3B hepatoma cells or in the murine hepatocyte cell line AML12, activin βE reduced the cell number and increased apoptosis rates [61,62]. Moreover, transient overexpression of βE by non-viral gene transfer in mouse liver inhibited regenerative DNA synthesis [60].

These observations suggest that activin E may have a growth limiting function similar to activin A [69]. However, the apoptotic function of the βE subunit in human endometrial tissue remains unclear. The observed increase in immunohistochemical staining of this subunit during late secretory phase is similar to the increase found for the βA-and βB-subunits [11] and suggests they all could have a similar function. However, the functions of these novel subunits and the formation of putative inhibins and/or activins warrants further research.

## Conclusions

In conclusion, the inhibin βC- and βE-subunits were expressed by normal human endometrial tissue. The immunolabeling of inhibin βE in human endometrium varied with the different stages of the menstrual cycle while the synthesis of the βC-subunit did not. Although the results show that epithelial cells are the predominant source of these subunits in normal endometrium, they were also detected in endometrial stroma throughout the menstrual cycle. The differential expression pattern of these β-subunits suggests that they have an important function in endometrial maturation and blastocyst implantation. Moreover, they probably participate in the paracrine signaling necessary for adequate endometrial maturation.

## Competing interests

The authors declare that they have no competing interests.

## Authors' contributions

IM and MSK carried out the data collection and data analysis, and drafted the manuscript. The study was conceived by IM, who performed also the statistical analysis. AB participated in the design of the study and performed the PCR analysis. IM and NS participated in the histological evaluation of endometrial samples. SK performed the immunohistochemistry for all inhibin-subunits. All authors have read and approved the final manuscript.
