# Correction: Evidence of inhibin/activin subunit betaC and betaE synthesis in normal human endometrial tissue

**DOI:** 10.1186/1477-7827-9-1

**Published:** 2011-01-05

**Authors:** Ioannis Mylonas, Ansgar Bruning, Naim Shabani, Susanne Kunze, Markus S Kupka

**Affiliations:** 1 Ludwig-Maximilians-University Munich, 1st Department of Obstetrics and Gynecology, Maistrasse 11, 80337 Munich, Germany; 2 Department of Obstetrics and Gynecology, Klinikum Neuperlach, Munich, Germany

## Correction

We have previously demonstrated the expression of inhibin subunits in endometrial tissues and used β-actin primer pairs to perform a PCR analysis loading control [1]. However, instead of using the mentioned β-actin primer pairs from a commercial molecular biology supplier, we used the β-actin primer pair listed in Table 1. All depicted primer pairs in Table 1 were designed and established by our group, and synthesized by the supplier Biomers.net (Ulm, Germany).

**Table 1:** Primer sequences and length of amplification products

	Forward primer (5' - 3')	Backward primer (5' - 3')	Length
**Inhibin-α**	CCGGCCATCCCAGCATACACGC	GAGTTGAGCGTCGGGCTCTC	359 bp
**Inhibin-βA**	TGCCCTTGCTTTGGCTGAGA	ACTTTGCCCACATGAAGCTTT	282 bp
**Inhibin-βB**	GGCGAGCGGCGACTCAACCTAGA	CGTGTGGAAGGAGGAGGCAGAGC	333 bp
**Inhibin-βC**	GCAGCCCGGGTGAGAGTTGG	ACTGCACCCACAGGCCTC	393 bp
**Inhibin-βE**	AGCCCTTCCTAGAGCTTAAG	GCTGCAGCCACAGGCC	404 bp
**β-actin**	GGAGAAGCTGTGCTACGTCG	CGCTCAGGAGGAGCAATGAT	366 bp

bp = base pairs
